# Fungal Pigments: Potential Coloring Compounds for Wide Ranging Applications in Textile Dyeing

**DOI:** 10.3390/jof6020068

**Published:** 2020-05-20

**Authors:** Chidambaram Kulandaisamy Venil, Palanivel Velmurugan, Laurent Dufossé, Ponnuswamy Renuka Devi, Arumugam Veera Ravi

**Affiliations:** 1 Department of Biotechnology, Anna University, Regional Campus – Coimbatore, Coimbatore 641046, Tamil Nadu, India; renukadev@gmail.com; 2 Department of Biotechnology, Alagappa University – Science Campus, Karaikudi 630003, Tamil Nadu, India; palanivelmurugan2008@gmail.com (P.V.); aveeraravi@rediffmail.com (A.V.R.); 3 CHEMBIOPRO Chimie et Biotechnologie des Produits Naturels, ESIROI Département agroalimentaire, Université de la Réunion, F-97490 Sainte-Clotilde, Ile de La Réunion, Indian Ocean, France

**Keywords:** fungal pigments, textile dyeing, toxicity testing, biotechnological approaches, challenges, limits

## Abstract

Synthetic pigments/non-renewable coloring sources used normally in the textile industry release toxic substances into the environment, causing perilous ecological challenges. To be safer from such challenges of synthetic colorants, academia and industries have explored the use of natural colorants such as microbial pigments. Such explorations have created a fervent interest among textile stakeholders to undertake the dyeing of textile fabrics, especially with fungal pigments. The biodegradable and sustainable production of natural colorants from fungal sources stand as being comparatively advantageous to synthetic dyes. The prospective scope of fungal pigments has emerged in the opening of many new avenues in textile colorants for wide ranging applications. Applying the biotechnological processes, fungal pigments like carotenoids, melanins, flavins, phenazines, quinones, monascins, violacein, indigo, etc. could be extracted on an industrial scale. This review appraises the studies and applications of various fungal pigments in dyeing textile fabrics and is furthermore shedding light on the importance of toxicity testing, genetic manipulations of fungal pigments, and their future perspectives under biotechnological approaches.

## 1. Introduction

Rapid industrialization in modern times has pressed the swift formulation and use of synthetic colorants in increased volumes in the food, medical, textile, and other industries regardless of their carcinogenic, immunosuppressive, and non-eco-friendly effects. Presently, many studies are focusing on finding alternatives to synthetic colorants, thereby improving the quality of the environment that affects various life forms. Nevertheless, advancements in biotechnology and the widespread awareness in ecological conservation, environmental protection, healthcare, etc. have generated a fervent interest among the public, industry, and researchers for exploring colorants from natural resources as an alternative to synthetic colorants.

Color plays a vibrant role in product acceptability in several industrial segments [1]. Dyes and pigments provide coloring ingredients and have been exploited by man for their artistic value. Dyes, being much smaller than pigments, are easily soluble during application and lose their nature whereas pigments, being about 1–2 μm, are insoluble. Moreover, pigments and dyes differ only by physical characteristics and not by chemical characteristics [2]. The dyes adhere to the surface and form covalent bonds with salts or metals by either physical adsorption or mechanical retention. Dyes used in the textile industries are carcinogenic and may trigger allergic reactions, and they have an environmental limitation because these dyes require strong acids, alkalis, solvents, and heavy metal catalysts, leading to toxic reactions.

Textiles is the key industry, among other industries, that uses diversified dyestuff. Lebeau et al. [3] reports that the anguish dyestuff industry requires higher natural pigments that have led to the emergence of natural colorants from microbes [4]. The increasing demand for pigments promotes multiplying research activities to explore filamentous fungi for pigment production. Research studies have shown that eco-friendly natural pigments from fungi are the best alternative to synthetic pigments because of their fast growth, easy processing, and important roles in transcription and intercellular communication. Fungal pigments have resistance and protect against biotic and abiotic agents (antagonistic microbes and UV radiation) and possess various biological applications [5]. Fungal pigments also have possible opportunities for commercializing their pigmented biomolecules for their application in food, cosmetics, textile fabrics, etc., because of their versatility, structure, and ease for large scale cultivation [6].

The present review appraises the research status of fungal pigments and their applicability in textile fabrics across broad characteristics like the ecology and diversity of fungal pigments, toxicity testing, biotechnological approaches for enhanced production, etc. Moreover, it aims to draw the attention of the academic and textile industries toward the challenges and the application of fungal pigments for textile dyeing, besides the prospective of genetic engineering research avenues in this field.

## 2. Historical Note on Pigments

The art of coloring has spread right from the development of human civilization. The use of pigments as coloring agents has been in vogue since prehistoric times. Europe and China, more than 5000 years ago, practiced dyeing with plants, leaves, barks, and insects. The Indus Valley-era, as early as 2600–1900 BCE (Before the Common Era or Before the Current Era), used madder dye to color garments at Mohenjodaro and Harappa. Roman centurions extracted red colorants from marine molluscs, *Murex* sp. to dye tunics and Egyptians used natural indigo from the plant *Isatis tinctoria.* Chinese Yanghai used alizarin, purpurin, and indigo in textiles in the Late Bronze Age (1700 BC). During the same Bronze Age, Phoenicians extracted Tyrian purple from the murex shellfish; though it was expensive, it was highly looked-for and so this colorant was the first initiation to global trade [7]. Generally, natural dyes have a strong tradition in India, Turkey, Mexico, Morocco, Europe, China, and countries of West Africa. Thus, natural dyes and dyestuffs are closely associated right from human civilization and are as old as textiles.

Dyes from insects (cochineal and kermes) were common in the 15th century. Cochineal (crimson) dye from cactus insects was in use to dye clothes and as an artist’s pigment, and later in food items. France, Holland, and Germany started using plant dyes in industry in the 16th century, and England used wood to dye clothes in the 17th century. Following the gradual application of natural dyes in different parts of the world, quercitron, the pigment extracted from the inner bark of oak, was patented as a dyeing material in America in 1775 [8].

Later, in the 18th century, Swedish chemist, Scheele discovered that chlorine destroyed vegetable colors, and following that, indigo began to develop in England. A natural dye, cudbear, which was extracted from various lichen sources, was later patented. The use of saffron from plants and cochineal from animals to dye clothes have been reported, and natural colorants from plants and animals were used until the middle of the 19th century [9]. About 95% of plants were characterized for plant pigments in America and Europe, however, due to several drawbacks to extract color from plants (stability, shelf life, etc.), researchers in the dye stuff industries looked forward to other alternate sources for colorants with better stability and shelf life.

In 1856, as an alternative to natural colorants, William Henry Perkin, a British chemist, discovered the first synthetic dye ‘mauve’ from artificial quinine. It was a favorite color of the royal family and so its importance consequently promoted the bloom of the innovative synthetic colorant industries. Thereafter, the industrial revolution rapidly propelled the production of synthetic colorants, which attracted various markets due to the easy manufacturing processes and production of superior coloring properties with less cost. Commercial dyeing industries, appreciating the undercurrent of the industrial revolution, switched over to synthetic dyes due to their production advantages and market potential, and consequently to date, synthetic colorants rule these industries, especially in the textile industry.

## 3. Ecology of Fungal Pigments

Fungi are diverse and abundant eukaryotic organisms on earth and their presence, even in extreme ecosystems, make it possible for them to produce novel secondary metabolites. Fungi inhabiting a plethora of ecosystems from terrestrial milieus to marine environments are omnipresent. They are spread across various eco zones, from polar to tropical regions and from aerial to deep-sea environments [10]. Current studies have reported the production of new molecules, mainly new pigments from a marine environment. Several polyketide compounds with novel biological activity have been isolated from fungi in deep-sea environments [11]. Marine ecological niches are still mostly unexplored and characteristics of marine ecosystems like salinity, low temperature, and dark induce microbes to produce novel metabolites. Tropical ecosystems are potential niches, and it is mainly mangroves that have the highest diversity of marine fungi because of their rich organic matter, which favors the production of valuable metabolites. Researchers have found that in extreme conditions, pigmented fungi could tolerate hydration/dehydration cycles and high radiation better than non-pigmented fungi. For instance, fungal melanin produced by many filamentous fungi has antioxidant activity, thereby protecting the structures, and provide durability to survive in aggressive environments. Hirot et al. [12] reported a novel metabolite, a green pigment, terphenylquinone, from *A. niger* isolated from Mediterranean sponge, *Axinella damicornis.*

## 4. Fungal Pigments

**Figure 1:**
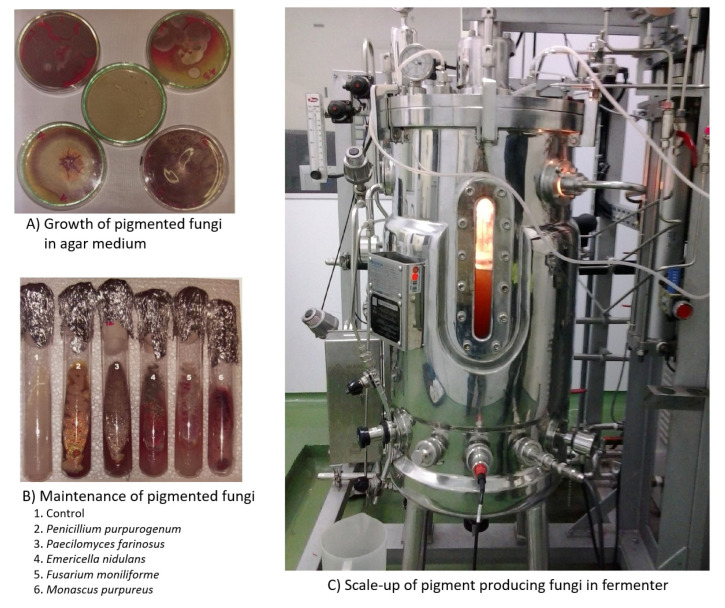
From Petri dish to fermenter: scale-up of pigment producing fungi. (**A**) Growth of pigmented fungi in agar medium; (**B**) Maintenance of pigmented fungi; (**C**) Scale-up of pigment producing fungi in fermenter.

Filamentous fungi produce amazing pigments like carotenoids, melanins, flavins, phenazines, quinones, and monascins from different chemical classes [13]. Carotenoids and polyketides come under the classification of fungal pigments [14]. Most fungi produce pigments that are water soluble and ideal for industrial production since it is easy to scale up in industrial fermenters and could be extracted easily without organic solvents (Figure 1).

### 4.1. Carotenoids

Carotenoids are formed through the isoprenoid pathway and produce a striking color with enormous biological activities like antioxidants, antimicrobials, membrane stabilizers, and precursors to vitamin A. Carotenoids are synthesized as carotenes (hydrocarbons) and xanthophylls (oxy-derivatives of carotenes). Several filamentous fungi produce different types of carotenoids (β-carotene, lycopene) and xanthophylls (astaxanthin, lutein, zeaxanthin, and violaxanthin), which are used in the animal feed industry for coloration and serve important roles as precursors of vitamin A.

Carotenoids are comprised mainly of C_40_ isoprenoids containing a polyene chain of conjugated double bonds [15]. They are lipid soluble pigments and have an aliphatic polyene chain that includes a conjugated double bond that acts as a chromophore and gives a yellow to red color. Avalos and Limon [16] reported that this conjugated polyene chain gives a chemical reaction against oxidizing agents and damage cell components. The carotene molecule is formed by head-to-head condensation of two geranylgeranyl precursors with acyclic C_40_H_56_ structures. Carotenoids possess ecological functions and protect against lethal photo-oxidation [17].

Fungal carotenoids are biosynthesized by the mevalonate pathway by 5-carbon isopentenyl pyrophosphate (IPP) as a precursor, synthesized from hydroxymethylglutaryl coenzyme A (HMG-CoA) or from derivatives of 1-deoxy-D-xylulose 5-phosphate (DXP) or 2-C-methyl-D-erythritol (MEP) generated from the condensation of pyruvate and glyceraldehyde 3-phosphate (non-mevalonate pathway) [18]. The IPP is condensed to form geranylgeranyl pyrophosphate (GGPP), which induces the formation of cis-phytoene. The introduction of α-/β ionone of the polyene chain gives the characteristic carotenoid structures. The MEP pathway starts from the condensation of pyruvate and glyceraldehyde-3-phosphate and is catalyzed by DXP synthase to produce DXP, consequently reduced into MEP by DXP reductase [19]. Avalos and Limon [16] reported that during the growth phase of *Penicillium* and *Phycomyces,* carotenoids are produced to protect from photo-oxidation. Bhosale et al. [20] reported that the cell functions are satisfied by sterols, dolichols, and ubiquinones, whereas as a response to environmental stress, astaxanthin and canthaxanthin are accumulated.

Ogbonna [21] reported that *Blakeslea trispora* produced β-carotene and lycopene; *Ashbya gossypii* gives lactoflavin (riboflavin), and *Penicillium oxalicum* produces a commercial colorant Arpink Red. β-carotene has been described in *Rhodosporidium* sp., *Penicillium* sp., *Aspergillus giganteus*, *Sclerotium rolfsii*, *Sclerotinia sclerotiorum*, and *Sporidiobolus pararoseus.*

### 4.2. Polyketides

Filamentous fungi produce more remarkable stable pigments than any other natural pigments [22]. Fungal polyketides are synthesized by polyketide synthases (PKS) from acetyl coA and malonyl CoA. Fungal polyketides are tetraketides and octoketides possessing eight C_2_ units. Polyketides represent anthraquinones, hydroxyanthraquinones, naphthoquinones, and azaphilones. Polyketide based fungal pigments produce a wide spectrum of colors ranging from red, yellow, orange, brown, and black. These polyketides contain valuable bioactive properties like anticancer, immunosuppressors, antimicrobials, antibiotics, etc. [23]. Many fungal polyketide pigments including anthraquinone, hydroxyanthraquinones, and naphthoquinones produce a wide range of colors for industrial applications. Anthraquinones are produced by *Fusarium* sp., *Trichoderma*, *Aspergillus*, *Eurotium* sp., *Penicillium* sp., etc. Arpink Red^TM^ (*Penicillium oxalicum*) was the first commercial product marketed by Ascolor Biotech, Czech Republic [13].

The derivative of anthraquinone is hydroxyanthraquinone with the replacement of one hydrogen atom by hydroxyl groups. *Aspergillus* sp., *Fusarium* sp., *Penicillium* sp., etc. produce hydroxyanthraquinone as intermediate metabolites [24]. Naphthoquinones are mainly produced by *Fusarium* sp., possessing yellow, orange, and brown colors [25]. Azaphilones are synthesized via the polyketide pathway and are produced by *Aspergillus*, *Chaetomium*, *Monascus*, and *Penicillium* [26]. Azaphilones have similar chemical and molecular structures like *Monascus* pigments [27]. *Monascus* produce six types of polyketide pigments: monascin (yellow), ankaflavin (orange), monascorubin, rubropunctatin, monascorubramine, rubropuntamine (red), and these pigments are sensitive to heat, light, and pH. *Monascus* pigments react with chemicals in the medium (proteins, amino acids, etc.) and form water-soluble pigments [27]. *Monascus* sp. produce edible pigments like monacolins, dimerumic acid, ergosterol, and γ-aminobutyric acid [28]. *Monascus* azaphilone pigments belong to yellow (monascin 1 and ankaflavin), orange (rubropunctatin 3 and monascorubrin 4), and red (rubropunctamine 5 and monascorubramine 6), respectively, and are produced in the genera *Monascus* and *Talaromyces* [29,30]. *Monascus* azaphilone pigments were used as additives in cosmetics because of their pleasant color and excellent capability to absorb harmful UV rays [28] and as dyes for printers, textile yarn, and also to improve the efficiency of solar panels when this pigment is applied as a novel sensitizing dye in solar cells [28,29]. *Monascus* azaphilone pigments have biological activities like anti-diabetic, anti-inflammatory, anti-cancer, anti-microbial, and anti-obesity properties [29,31]. *Monascus* sp. also produces monacolin K (lovastatin) and are used as serum cholesterol lowering drugs because of their inhibition toward 3-hydroxy-3-methylglutaryl-coenzyme A reductase, which controls the biosynthesis of cholesterol [32].

In spite of its tremendous economic potential, the biosynthetic pathway of *Monascus* azaphilone pigments is proposed. The orange pigments (rubropunctatin 3 and monascorubrin 4) were formed by the esterification of a β-ketoacid (fattyacid synthase pathway) to chromophore (polyketide synthase pathway) pathway, reducing the orange pigments to yellow pigments (monascin 1 and ankaflavin). On the contrary, the amination of the orange pigments with NH_3_ gives a red pigment (rubropunctamine 5 and monascorubramine 6) [33]. In addition to the typical *Monascus* azaphilone pigments, more than 100 of its congeners have been identified recently, which includes yellow (49), red (47), orange (8), and purple (1) pigments isolated from *Monascus* sp. and *Talaromyces (Penicillium)* sp. [30]. The structural diversity of these pigments is based on the number of carbon skeletons produced by core enzymes (polyketide synthases and non-ribosomal peptide synthetases) [34]. These carbon skeletons are modified by tailoring enzymes for various industrial applications, and metabolic engineering will ensure the safety and productivity of the fermentations.

### 4.3. Anthraquinones

Anthraquinones are found in *Aspergillus* sp., *Eurotium* sp., *Emericella* sp., *Fusarium* sp., *Penicillium* sp., *Mycosphaerella* sp., *Microsporum* sp., and exhibit a wide range of biological activities including antimicrobial, herbicidal, and insecticidal properties [35]. Anthraquinones produce a yellow color, whereas the substituents produce various hues of the molecules ranging from yellow, orange, red, brown, and violet. Anthraquinones are composed of three benzene rings with 1,10 dioxoanthracene (C_14_H_8_O_2_) and have two ketone groups in the center. Most widespread fungal anthraquinones are 1,8 dihydroxy and 1, 5,8 trihydroxy anthraquinone derivatives. Anthraquinones are present either in free form or glycoside attached to the O- or C- bond in the side chain, which makes them water-soluble. The characteristics of fungal anthraquinones are dimeric structures formed by C–C bonds. The three ring structure suggests that these compounds can intercalate with DNA and are used in small doses [36]. Another important characteristic is their absorption spectra at 405 nm [37]. They exhibited a wide range of colors with chromatic properties and is of interest for dyeing in the most requested industries like cosmetics, textile dyeing, printing, and food industries. Anthraquinones are quite complex, with a great diversity of chemical structure and parameters (light, pH, temperature, oxygen transfer, carbon and nitrogen sources, inoculum concentration, etc.), which have a large impact on pigment production.

### 4.4. Hydroxyanthraquinone

Hydroxyanthraquinone isolated from *Haloresellinia* sp. (marine fungus) showed cytotoxic activities [38]. Another hydroxyanthraquinone, aspergillus H and I from *Aspergillus versicolor* have exhibited antiviral activity toward HSV-1 [39]. Shi et al. [40] reported two new hydroxyanthraquinones, namely harzianumnones A (1) and B (2), from the coral fungus *T. harzianum*, possessing cytotoxic activity against the HeLa cell line.

Anthraquinones represent a class of the quinone family with a basic structure of 9,10-dioxoanthracene containing two ketone groups on a central ring. The diversity of these compounds depends on the position of the substituents replacing H atoms on the basic structure. When *n-*hydrogen atoms are replaced by hydroxyl groups, the molecule is called hydroxyanthraquinone. 1,3,6,8-tetra-hydroxyanthraquinones were isolated from *Microsphaeropsis* sp. (associated with marine sponge), *Geosmithia* sp., *Trichoderma* sp., and *Verticicladiella* sp. [37]. Hydroxyanthraquinone derivatives were isolated from the mangrove fungi *Guignardia* sp. and *Halorosellinia* sp., possessing cytotoxicity against the cancer cell line [41]. Fouillaud et al. [37] reported that the 1-hydroxy-3-methylanthraquinone containing only one hydroxyl group on the R1 position had excellent cytotoxic activity against cancer cells, whereas dihydroxyanthraquinones with 1-hydroxy decreased the anticancer activity. 1,4,6,8-tetrahydroxyanthraquinones from *Aspergillus glaucus* showed excellent antibacterial activity against *Bacillus brevis*. The hydroxyanthraquinone derivatives like 1,3,8-trihydroxy-6-methyl-anthraquinone, aloe-emodin 8-O-glucopyranoside, 1,8-dihydroxy-3-methoxy-6-methyl-anthraquinone, and 1,4,5-trihdroxy-7-ethoxy-2-methyl-anthraquinone were isolated from *Drechslera rostrate* and *Eurotium tonpholium,* possessing anti-leishmanial activity [42].

Hydroxyanthraquinone pigments have excellent light fastness properties and no metallization required for dyeing and are considered as reactive dyes. They form complexes with various metals viz aluminum, barium, calcium, copper, iron, etc. and display excellent brightness compared to azo dyes. The ability to form this type of complex is of great concern in textile industries because they easily form covalent bonds with many fibers like cotton, wool, and nylon [37].

### 4.5. Naphthoquinones

Among the quinones, naphthoquinones are important secondary metabolites and more than 100 naphthoquinones have been identified with different structures in filamentous fungi [43,44], exhibiting various activities like anti-microbial, anti-inflammatory, and anti-cancer properties because of their tendency to prevent DNA damage [45,46]. Newman and Townsend [44] reported that naphthoquinones are considered as the model system to study the synthesis of polyketides in filamentous fungi. On the contrary, Manicilla et al. [45] demonstrated that a significant difference exists in naphthoquinone biosynthesis, representing the pathway as polyphyletic and not polyketide. The 1, 4-naphthoquinones congeners with *Monascus* azaphilone pigments are referred to as *Monascus* naphthoquinones and are detectable in trace quantities only while fermenting *Monascus* azaphilone pigments by the wild-type of *Monascus* sp. [46].

Naphthoquinones are widespread in fungi with phytotoxic, antimicrobials, insecticidal, anti-carcinogneic, cytostatic activities, etc. Naphthoquinones are produced by *Chlorociboria* sp. and *Arthrographis cuboidea* and they differ from naphthoquinones produced by *Trichoderma* sp. and *Fusarium* sp., possessing antagonistic properties toward insects, bacteria, and fungi [47]. Naphthoquinone-like pigments are produced by wood spalting fungi *Sctalidium cuboideum* (draconin-red, red pigment) and *Chlorociboria* sp. (xylindein, blue-green pigment) [48]. Among these naphthoquinone-like pigments, xylindein has been researched for solar energy and textile dyeing applications and when used in the textile and paint industry as dyestuffs, draconin-red crystals formed with excellent color stability.

More than 100 types of naphthoquinone metabolites are produced by 63 fungal species [49]. Naphthoquinones are closely related to chemical defense and have biological roles in electron transfer in various oxidative process (photosynthesis, oxidative phosphorylation) [50]. They possess antifungal activity against all *Candida* sp. (*C. albicans, C. krusei, C. kefyr, C. parapsilosis*), a multi drug resistant pathogen, among bone marrow patients and is resistant to fluconazole [51]. Ferreira et al. [52] reported that the chemistry behind the mechanism of action should be understood.

### 4.6. Azaphilones

Azaphilones are interesting secondary metabolites with a structurally diverse class, having pyrone-quinone structures possessing high-oxygenated bicyclic core and chiral quaternary center. The biosynthesis includes the fatty acid synthesis pathway and polyketide pathway. The first pathway assembles the polyketide chain from acetic acid and five malonic acid to form the chromphore structures. The second pathway produces medium chain fatty acids by the transesterification reaction.

Azaphilones, a fungal polyketide containing a pyrone-quinone bicyclic core and quaternary carbon center, have various activities like anti-microbial, anti-oxidative, anti-cancer, and anti-inflammatory properties [53,54,55]. Penicilones A-D, novel azophilones with different configurations at the quaternary carbon center, has remarkable anti-MRSA activity [56]. Citrifurans A-D, unusual dimers of azaphilones, has an inhibitory activity against LPS-induced NO production [57]. Recently, 13 different types of azaphilones are classified based on their chemical structure, which includes the austdiol-type, bulgarialactone-type, citrinin-type, deflectin-type, hydrogenated spiro-azaphilones, and O-containing *Monascus* pigments [54] obtained from *Penicillium* sp. and *Talaromyces* sp. with a wide range of biological activities.

Austdiol-type azaphilones are characterized by an austdiol core with 19 members that include fusaraisochromenone, nemanecins A-C, and perangustols A and B [58,59]. Felinone A from *Beauveria feline,* xylariphilone from *Xylariales* sp., and aspergillusone C from *Aspergillus clavatus* showed cytotoxicity against various cancer cell lines [60,61,62]. Bulgarialactone-type azaphilones, 5,6-dihydroxyacetosellin and monakaocinol, have a conjugated and linear γ-lactone ring. These types of azaphilones have been isolated from marine fungus, namely *Epicoccum nigrum* and *Monascus kaoliang* [63,64]. Citrinin-type azaphilones are monomeric citrinin derivatives like annulohypoxylomans A-C, annulohypoxylomanols A-B, annulohypoxyloside, and pentaketide [65]. The novel citrinin analogues (3S,4R)-6-hydroxy-8-methoxy-3,5-dimethyl isochromanol and (3S)-6-hydroxy-8-methoxy-3- methylisochroman were isolated from *Penicillium* sp. [66]. The isochroman glycoside metabolites, monascuspilorin and monascupurpurin were produced by *Monascus pilosus* BCRC38072 and *Monascus purpureus* BCRC 31499 [67,68]. Penicitol A, a citrinin dimer from mangrove fungus *Penicillium chrysogenum* HND-11 has cytotoxic activity against HeLa, HEK-293, HCT-116, and A549 cell lines [69]. Deflectin-type azaphilones were characterized with a γ-lactone ring together with a ketone aliphatic chain [70]. Six deflectins from *Aspergillus deflectus* NCC0415 showed inhibitory activity against protein tyrosine phosphatases and SHP2. Colletotrichones from endophytic fungus *Colletotrichum* sp. exhibit remarkable antibacterial activity [71]. Coniellins from goose dung fungus, *Coniella fragariae,* showed inhibition of NF-kB activation in MDA-MB-231, thereby reducing the migration of tumor cells with more than 60% inhibition [70]. Hydrogenated spiro-azaphilones have a five- or six-membered ring spiroketal system on an azaphilone skeleton. Monascuspirolides A and B from *Monascus purpureus* BCRC 38110 showed stronger NO inhibitory activity with the IC_50_ value of 17.5 and 23.5 µM, respectively [72]. Peniazaphilin A from *Penicillium* sp. CPCC400786 has anti-HIV activity [73]. O-containing *Monascus* pigments has four azaphilones obtained from *Monascus* sp. Monascusazaphilones A and B from *Monascus purpureus* BCRC inhibited NO production by macrophages and it was stronger than the positive control [53].

Azaphilones are produced mainly by *Penicillium, Monascus, Chaetomium*, and *Talaromyces* exhibiting wide color tones as yellow, orange, and red. Xiong et al. [74] reported that the red color has been associated by reacting yellow pigments with amine groups by exchanging pyran oxygen for nitrogen. Azaphilones have a broad spectrum of applications in medicine like decreasing blood pressure, antioxidants, anti-microbials, etc. [75]. Osmanova et al. [55] reported that the activities are related to the reactions of azaphilones with the amino group in the formation of vinylogous γ pyridines.

## 5. Fungal Dyes for Textile Applications

The textile industry is the largest industry by economic contribution and employment generation. Fungal pigments have excellent colorfastness and staining properties and they are of great interest to textile industries; they warrant production under controlled conditions, with no seasonal fluctuations, and are biodegradable [14]. The burden of reducing the hazards to the environment rests with the dyeing industries. In spite of the positive features of fungal dyes, it has not met the expectations of the textile industry because of irregular fixation [76]. In addition, there are no standardized methods for the industrial dyeing of fungal dyes [48,77]. Therefore, a proper method to standardize the industrial dyeing of fungal dyes and many novel fungal pigments should be taken forward for standardized industrial applications.

Fungal pigments, due to their stability and consistency, have been reported for their use as alternatives to synthetic dyes in the textile industry [47]. These pigments can absorb light in the ultraviolet region and when applied as textile dyes, can protect human skin from harmful UV radiation [78] (Nambela et al. 2020). The potential of fungal pigments in the textile industries has been investigated by various researchers [79,80,81,82]. Through a biotechnological approach, anthraquinones are produced by fungi, namely *Trichoderma* sp., *Drechslera* sp., *Aspergillus* sp., and *Curvularia* sp. Cynodontin extracted from *Curvularia lunata* successfully produced two anthraquinone dyes similar to Disperse blue 7 and Acid green 28, the characteristics of which are similar to conventional dyes [83].

Anthraquinones have been reported to be produced from various fungi with antimicrobial activities. Anthraquinones from *Sclerotinia* sp. produce a pink shade and dyed cotton yarns with chemical and natural mordants. The yarns showed excellent stability to heat, light, pH, and temperature [84]. Osmanova et al. [55] reported the existence of water-soluble red and yellow pigments from *T. australis* and *P. murcianum* with an affinity toward wool. Results of this study indicated that the dyes were suitable for industrial conditions as they could withstand temperature and pH. Chemical analysis reported that the red dyes were similar to *Monascus* type pigments [85]. *Penicillium* sp. produces ankaflavin (*Monascus* type pigments) and the ionic nature has a strong affinity of dyes with wool [86]. Nagia and El-Mohamedy [87] reported that anthraquinones from *Fusarium oxysporum* could be used for dyeing wool with excellent color fastness properties and high dye uptake. Anthraquinones produce bright hues with excellent fastness properties and chemical modification may be interesting if it could facilitate the synthesis of dye molecules.

Morales-Oyervides et al. [88] reported the potential application of pigment from *Talaromyces* sp. in the textile industry. The results confirmed the pigment to be a novel source to dye wool textiles. High pigment uptake by the fabric was observed, and the dyeing rate constant and half time dyeing with kinetic behavior well matched those of natural dyes used in the textile industry. The *Talaromyces* pigment has potent antimicrobial properties coupled with the absence of toxicity, which makes this pigment a valuable alternative as a natural dye in textile dyeing [89]. Further characterization of the molecule and study on the interactions between the dye and fabric are worth future research. Celestino et al. [90] reported pigmented fungi *Penicillium sclerotiorum* 2AV2, *Penicillium sclerotiorum* 2AV6, *Aspergillus calidoustus* 4BV13, *Penicillium citrinum* 2AV18, and *Penicillium purpurogenum* 2BV41. *P. sclerotiorum* 2AV2 from soil from the Amazon produces intense color pigments, which could be used for textile applications.

Hinsch et al. [91] reported that the fungal pigments isolated from rotting hardwood logs in Canada produced xylindein (green pigment from *C. aeruginosa*), draconin red (red pigment from *S. cuboideum*), and yellow pigment (*S. ganodermophthorum*) and were able to dye multi fabric test strips. Their results indicated that all these pigments could be used to dye fabrics without the need for additional chemicals. Xylindein exhibited good potential to dye garment fabrics and draconin red for second layer garment fabrics. Spalting (wood-rotting) fungi have been the hot topic of research to extract novel pigments for textile applications. Awkwardly, dichloromethane (DCM) is used to extract colorants from spalting fungi and causes environmental problems and health issues, which is one of the major hurdles holding back spalting fungi from commercialization. Researchers have found that natural oils could be used to transfer pigments from *Chlorociboria* sp., *Scytalidum cuboideum*, and *Scytalidium ganodermophthorum* onto host substrates [92].

**Figure 2:**
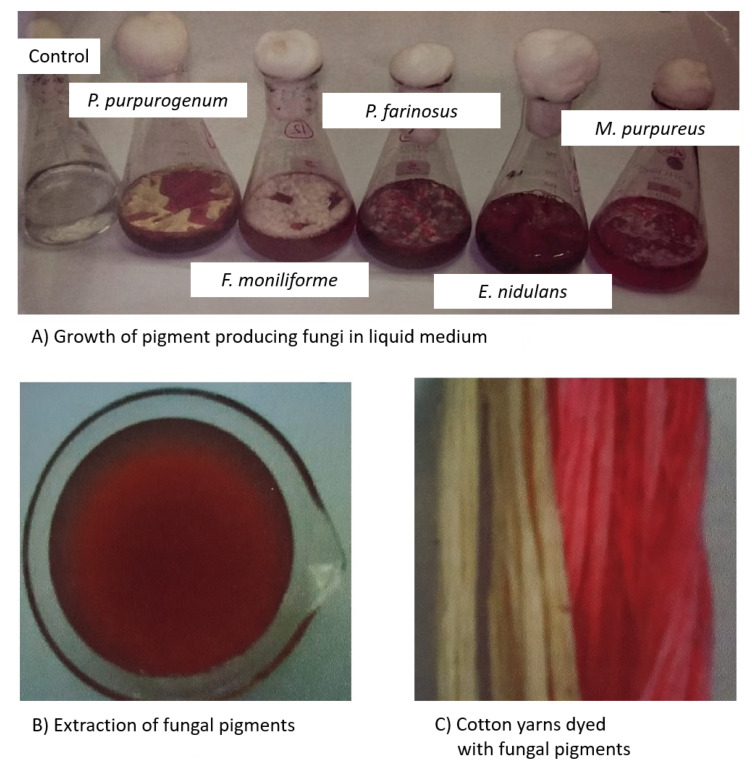
Water-soluble fungal pigments for dyeing cotton yarns. (**A**) Growth of pigment producing fungi in liquid medium; (**B**) Extraction of fungal pigments; (**C**) Cotton yarns dyed with fungal pigments.

Gupta et al. [93] attempted to isolate pigment from *Trichoderma* sp. and explored the possibility for dyeing silk and wool fabrics. The dye from *Trichoderma* was non-toxic to human skin and possesses antimicrobial properties. The color value for the dyed sample was higher for wool when compared to silk fabric, and showed excellent fastness properties to washing and rubbing. Poorniammal et al. [94] evaluated the yellow pigment from *Thermomyces* sp. for the textile dyeing process. Natural mordants and the yellow pigment reduced the influence of pathogens. The overall color fastness properties for the dyed silk fabric was moderate. Due to these antimicrobial properties, this can be used especially in medical applications like bandages, masks, wound dressings, etc. Devi and Karuppan [95] reported on reddish brown pigments from *Alternaria alternata* for their efficiency to dye cotton fabrics. The pigments showed good color fastness properties to perspiration and rubbing. This was the first report to study the reddish brown pigments for *Alternaria alternata* for dyeing cotton fabrics. Velmurugan et al. [96] assessed different water-soluble fungal pigments from *Monascus purpureus*, *Isaria farinosa*, *Emericella nidulans*, *Fusarium verticillioides*, and *Penicillium purpurogenum* for dyeing cotton yarns (Figure 2). This study testified that pre-mordanting with alum and ferrous sulfate achieved variation in shade and color.

The reddish-brown pigment from *Phymatotrichum* sp. (NRC 151) was produced using H-acid (1-naphthol-8 amino, 3,6-disulfonic acid) as a precursor in the medium that showed better fastness to washing, perspiration and light and can be used to dye various fabrics [24]. Sharma et al. [77] isolated *Trichoderma virens, Alternaria alternata*, and *Curvularia lunata* from different habitats for pigment production. The pigments applied to silk and wool showed good fastness properties. The optimum condition for maximum pigment production should be standardized for commercialization in an eco-friendly manner with a cost reduction.

**Figure 3:**
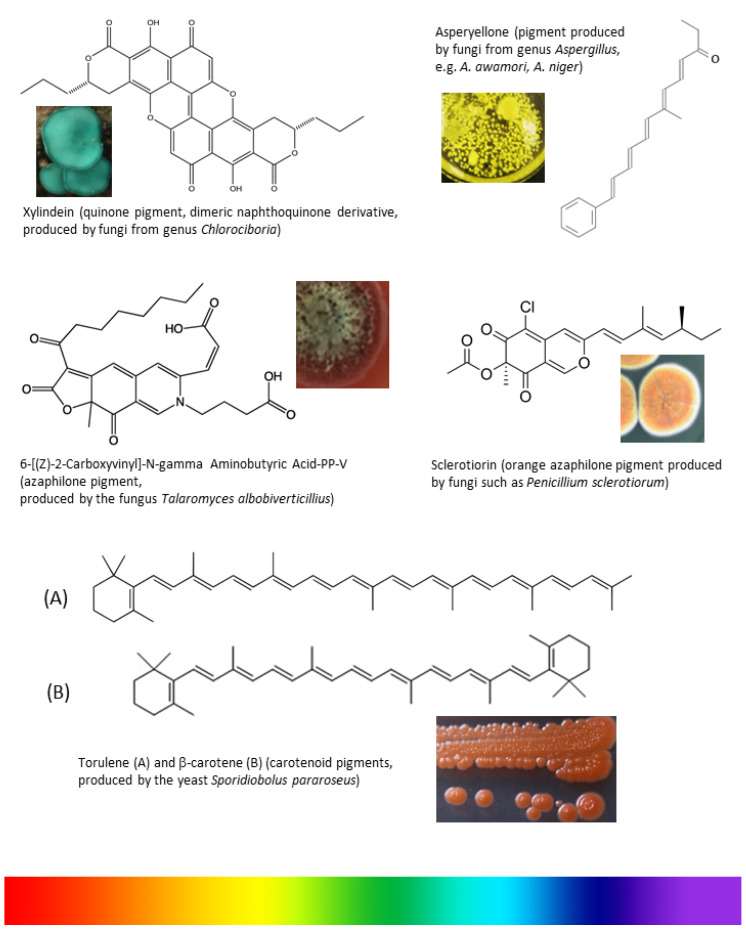
Chemical structures of fungal pigments with potential coloring properties that could be used in textile dyeing.

Weber et al. [48] isolated three eco-friendly fungal pigments from *S. cuboideum, C. aeruginosa*, and *S. ganodermophthorum*, which showed their textile dyeing capability without using water and thermal energy. The pigment showed no fading over one week’s time and further research is required to study the relationship between the pigments and fabrics, skin sensitivity, toxicity, and stability (UV, time, wash and wearing). Different fungal species with their active pigment for application in the textile industry are shown in Table 1 and Figure 3.

## 6. Toxicity Testing for Fungal Pigments

Heo et al. [105] studied the toxicity of fungal pigments extracted from *Penicillium miczynskii*, *Sanghuangporus baumii*, *Trichoderma* sp. 1, and *Trichoderma afroharzianum.* The pigments exhibited high radical-scavenging activity. Moderate toxicity was observed in *S. baumii* by the acute toxicity test limiting the applications of this pigment in industry. *P. miczynskii*, *Trichoderma* sp. 1, and *T. afroharzianum* were reported to be the best fungal pigment for producing strains for safe, water-soluble pigments in the industry.

The cytotoxic activity of fungal pigments from *F. oxysporum, T. verruculosus*, and *Chaetomium* sp. has been tested using various methods such as the yeast toxicity test (YTT), brine shrimp lethality bioassay, or cell counting kit-8 assay. This method of cytotoxicity assay warrants the application of pigments in various industries, especially in the health and pharmaceutical sectors [87]. Poorniammal et al. [106] studied the dermal toxicity of pigments from *Thermomyces* sp. and *P. purpurogenum* in Wistar rats and their results showed that the pigments were non-toxic and have broad scope in the dyeing, printing, and cosmetics industries.

The deep sea fungus *Chaetomium* sp. AN-S01-R1 produces chlorinated azaphilone pigments like chaephilone C (compound **1**) and chaetoviridides A-C (compounds **2**–**4**). Compound **2** exhibited potent cytotoxic activities toward HepG2 cells with IC_50_ below 5 µM, whereas compounds **1** and **3** showed stronger cytotoxic activities against HeLa cells [107]. To study the cytotoxic mechanism of pigments and their possible industrial applications, further research should be conducted.

The red pigment from *Talaromyces verruculosus* was used to dye cotton fabrics with excellent dye uptake and color fastness properties. The cytotoxicity assay conducted using the brine shrimp lethality test revealed insignificant toxicity and was harmless to use. Hence, researchers should focus on obtaining pigments from this strain for industrial applications and further chemical characterization will open new avenues in the dyeing industry that will reduce the adverse effects of synthetic dyes [104].

Pandiyarajan et al. [108] reported the presence of water-soluble yellow pigments from *Aspergillus* sp. The pigment possessed maximum dyeing ability of the fungus with the hydrothermal method for textile fabrics without mordants and showed better dye uptake in comparison with synthetic dyes. The toxicity of the pigment was tested using a zebra fish model system with an IC_50_ value of 710 µg/mL. This novel pigment can be used an alternative to synthetic dyes for applications in the textile industrial sector.

## 7. Biotechnology Ways for Enhanced Production

The improvement of fungal pigment production and the knowledge to improve the yield have been gradually studied and extended based on the following major criteria: (1) Through genetic manipulation, the pathways for pigment production and the molecules are better understood as many fungal genomes have been sequenced and many others are still in progress; (2) molecular screening techniques help to improve the gene expression and secretion of unusual metabolites; and (3) the use of optimization strategies such as the use of artificial intelligence to improve the fermentation conditions for pigment production.

Today, the higher production of synthetic dye based industries makes it challenging to use microbes at the industrial level. The major reason associated with this is the higher production cost for instruments, chemicals, and processing. The same can be sorted out using agro-industrial residues for the cheap production with same metabolites at the industrial level. Numerous research on fungal pigments has led to the robustness and tolerance against possible stress at the industrial level, which warrants the efficient production of fungal pigments.

### 7.1. Genetic Manipulation

The biosynthesis of pigment producing fungi has not been studied in detail and hence genomic screening for pigmented fungi is not possible at the premature stage. The classical methods such as taxonomy, biochemistry, and physiology will be very active in screening pigment producing fungi. The major breakthrough is to genetically modify the strain by genetic transformation, enabling scientists/researchers to modify the targeted gene for unusual metabolites. The genetic transformation of fungi is tedious because of its complex cell wall structures and lack of genetic markers. Hence, species-specific transformation must be required and optimized for every potent strain.

Using genetic engineering approaches like genetic modification, cloning of genes, and exclusion of non-essential genes (toxins), various research studies have been undertaken for enhancing fungal pigment production. Fungal carotenoids from native carotenogenic fungi, *Blakeslea trispora* and *Phycomyces blakesleeanus* are produced up to the industrial scale (17 grams of β-carotene per liter in some cases). Several attempts have been made to introduce the biosynthetic pathway of lycopene or β-carotene in non-carotenogenic strains using metabolic engineering methods. The enhancement of isopentenyl diphosphate (IPP) and dimethylallyl diphosphate (DMAPP) precursors of the MEP or MVA pathways have demonstrated the improved production of lycopene and β-carotene [109].

Fungal strains producing polyketides are not easily cultured in liquid fermentations due to their filamentous growth and dense mycelial growth, which result in increased viscosity and reduced oxygen transfer. The transfer of the polyketide biosynthetic pathway to an industrial strain such as *Saccharomyces cerevisiae* offers an attractive alternative as it allows for easier fermentation and process optimization by metabolic engineering strategies [110]. Type 1 polyketide synthases (PKS) play a vital role in the synthesis of fungal polyketides. The PKS are proteins and are related to eukaryotic fatty acid synthases. Acetyl coenzyme A and malonyl CoA condense to form carbon chains whereas ketoacyl CoA synthase, acyltransferase, acyl carrier domains are also required for the synthesis of polyketides [4]. In *Monascus*, three pigmented or not polyketides are known to be produced, namely citrinin, red pigment, and monacolin K [23]. Many approaches have been used to decrease the production of citrinin (mycotoxin), thereby increasing the production of red pigment. The optimization of various parameters like nitrogen sources, pH, dissolved oxygen, and important genetic alterations were employed to reduce the production of citrinin. In the industrial strain, *M. purpureus* SM001, the polyketide synthase gene *pksCT* disruption, successfully eliminated citrinin production [111].

The investigation about polyketide pigments produced by *F. graminearum, F. decemcellulare,* and *F. bulbigenum* and characterized as naphthoquinones explained that their biosynthesis was the response to environmental stress [112]. Aurofusarin, the main naphthoquinone produced by *Fusarium* sp., exhibited that the gene cluster *PKS12* was responsible for aurofusarin biosynthesis under the control of local transcription factor *AurR1* [113]. The *PKS12* gene cluster is also responsible for the production of rubrofusarin and fuscofusarin [114]. Rubrofusarin is an orange-brown pigment reported to inhibit human DNA topoisomerase II-α and has antibiotic effects on *Mycobacterium tuberculosis.* The polyketide core scaffold differs in their tailoring enzymes, resulting in the production of end products like rubrofusarin B, aurofusarin, nigerone, and nigerasperone A.

The yellow pigment chrysogine by *Penicillium chrysogenum* protects the fungi from ultraviolet radiation and lacks antimicrobial and anticancer activities [115]. The putative biosynthetic gene cluster identified in *P. chrysogenum* includes non-ribosomal peptide synthetase (NRPS) [116], and lately, Wollenberg et al. [117] proved that NRPS is responsible for chrysogine biosynthesis in *Fusarium graminearum.* Anthraquinones have been genetically modified using *Aspergillus* sp., expressing genes related to monodictyphenone and atrochrysone biosynthesis. The metabolic gene cluster for the biosynthesis of red pigment bikaverin from *Fusarium* sp. includes gene encoding PKS (*bik1*) and two genes (*bik2* and *bik3*) encoding tailoring enzymes as well as general transcriptional activator (*bik4*), specific transcriptional activator (*bik5*), and a transporter (*bik6*) [118].

Sen et al. [119] reported that various methods decreased the production of citrinin and mycotoxin, thereby increasing the production of red pigments. The polyketide synthase gene has been studied extensively for the synthesis of citrinin by *Monascus purpureus*. The polyketide synthase gene pksCT was effectively cloned to remove citrinin, thereby enhancing red pigment production by the industrial strain *M. purpureus* SM001 [111]. Lee et al. [120] enhanced the production of monacolin K by *M. pilosus* by *laeA* overexpression using an *A. nidulans alcA* promoter.

The metabolic engineering in fungi is extremely tedious due to the lack of genetic markers and low gene targeting frequencies. CRISPR (Clustered Regularly Interspaced Short Palindromic Repeats) has been successfully used to produce compounds of industrial importance. It consists of enzyme Cas9, the molecular scissors that makes a cut in the target location, enabling the addition or removal of pieces of DNA and guide RNA located inside a longer RNA scaffold. This scaffold binds to target DNA whereas the guide RNA directs the Cas9 enzyme to make a cut at the right point, activating the DNA repair mechanism, and can be used to introduce changes to one or more genomes [121]. Hence, this system is successfully used in genetic engineering to make cell factories for the cost efficient production of natural pigments [122].

Nielson et al. [123] studied the CRISPR cas9 system for *Aspergillus nidulans*, which can also be applied to other fungal types. This has been used for *Talaromyces atroroseus*, a red pigment producer targeted for the food industry. Pohl et al. [116] reported the use of CRISPR cas9 in *Penicillium chrysogenum* with improved production. Limited research is available for pigmented microbes and more research is needed to optimize this system for future industrial applications.

Jia et al. [111] studied the elimination of the mycotoxin citrinin by metabolic engineering. A binary vector system was constructed that disrupts the polyketide synthase gene pks CT in *M. purpureus* SM001 by *Agrobacterium tumefaciens* mediated transformation. The established system was evaluated and showed a high efficiency to improve industrial *Monascus* strains. Westphal et al. [113] reported the enhanced production of aurofusarin by *Fusarium graminearum* by examining the transcription factor AurR1 on the aurofusarin gene cluster in the strain. The overexpression of Aur R1 increased five proteins of the aurofusarin pathway, leading to the 3-fold increase in the production compared to the wild strain.

### 7.2. Agro-Waste for Clean-Up Production

**Figure 4:**
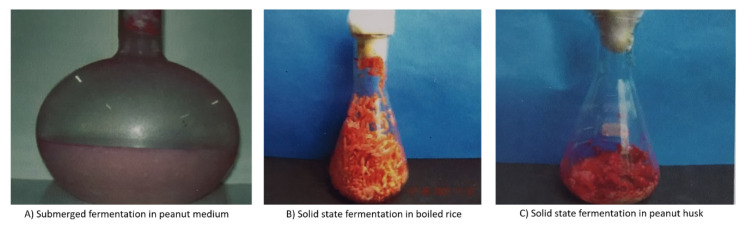
Growth of *Monascus* sp. in different agro substrates. (**A**) Submerged fermentation in peanut medium; (**B**) Solid state fermentation in boiled rice; (**C**) Solid state fermentation in peanut husk.

Agricultural activities produce waste (straw, stem, stalk, husk, peel, legumes, seeds, bagasse, spent grains etc.) throughout the year. There is a great interest in re-using these nutrient rich materials for bioprocessing. The use of this waste can act as a substrate for low cost raw materials to make the process for the production of value added products. Recently, raw materials and byproducts of agro-industrial residues have been considered as low cost substrates for pigment production by microbes (Figure 4). Lopes et al. [124] reported that different strains of filamentous fungi produced pigments on a cheese whey and soya protein medium. Similarly, Kaur et al. [125] studied the enhancement of yellowish pink pigment by *Rhodotorula rubra* MTCC 1446 in a whey and coconut water medium.

Subhasree et al. [126] stated that red pigment by *Monascus purpureus* was produced by utilizing jackfruit seed as a substrate, and another study reported the production of red pigment by utilizing durian seeds [127]. By utilizing grape waste as a growth substrate, red pigment production by *Monascus purpureusin* was optimized using the response surface methodology [128]. The production of carotenoids by *Rhodotorula glutinis* 1151 was optimized by RSM using a tomato waste medium. Sugarcane and corn bagasse have also been used for pigment production by *Monascus* sp. [129]. Taskin et al. [130] prepared a cheap peptone source by utilizing chicken feather waste through acid hydrolysis and investigated the suitability of this peptone source as a substrate for carotenoid production by *Rhodotorula glutinis* MT-5. Interestingly, 92 mg/L of carotenoids were produced, and this source could be used as a cheap substrate for carotenoid production.

The industries, as a beneficial measure to them, may utilize agro-industrial residues as inexpensive growth substrates for microorganisms. Consuming residues of agro-industries in a bioprocess reduces the costs of production, also solving pollution problem associated with their disposal. Lopes et al. [124] reported the readily available source for pigments produced from *Penicillium chrysogenum* IFL1 and IFL2, *Fusarium graminearum* IFL3, *Monascus purpureus NRRL* 1992, *Penicillium vasconiae* IFL4. All of the fungi produced the water-soluble pigments monascorubin, rubropunctatin, and mycotoxin citrinin on agro-industrial residues (feather meal, fishmeal, cheese whey, grape waste, soybean meal, and rice husk). Sanchez [131] reported the use of agro-industrial residues for low cost alternatives for pigment production, thereby reducing 73% of the total production cost. The main aim is to lower the cost by reducing the cost of raw materials and reducing environmental pollution.

## 8. Limits, Challenges, and Future Scope for the Dyeing of Fungal Pigments

The reporting of synthetic textile colorants as carcinogenic because of the presence of dioxins (polychlorinated dibenzo-p-dioxins and polychlorinated dibenzofurans) has resulted in the development of eco-friendly, non-toxic colorants. Fungal colorants from a cost effective process that display high colorfastness properties are promising. The authorized usage of fungal pigments varies in diverse parts of the world with respect to its local and traditional usage [13]. For example, the applications of *Monascus* like pigments from *Penicillium* sp. on textile, cotton, wool, leather, paper, paint, etc. have been patented by Mapari et al. [132]. Added important features include the enhanced production of pigments, solubility in water, and stable pigments [133]. The pigment yield can be enhanced by improving the fungal growth or by increasing the accumulation of pigments in the cells [134].

The main challenge is the difficulty in increasing both pigment and biomass yield, since both are negatively correlated. Hence, the relationship between biomass and pigment production needs to be thoroughly studied in order to produce pigments in a controlled way. Pigment production can be controlled by using genetic engineering techniques. Recombinant DNA technologies have been used to alter the activity of enzymes involved in the biosynthesis of carotenoids [135]. The challenges associated with the commercial production of fungal pigments (*Monascus* pigment) is its low solubility in water and poor stability to pH, heat, and light. Dufossé [136] reported that methods have been developed to address these challenges by replacing oxygen with nitrogen from the amino group in the pigment’s structure. Furthermore, the major problem is the co-production of toxic compounds with the pigment limiting its applications and preventing regulatory approval.

Most fungi produce a mixture of pigments and the challenge is to produce a pigment with one specific color tone. This can be achieved by optimizing the parameters like substrates, pH, temperature, dissolved oxygen either in solid state or in submerged fermentation. The prospect of developing fungal pigments at the industrial level by selecting the strain with safety measures is imperative. Cost-effective fungal pigments can be produced at lab scale and the technical hurdles that arise when designing the industrial plant need attention. Another important issue is the stability of the pigment over time.

The valuable metabolites from fungi are limited for its commercialization due to the production of mycotoxins together with pigments. Furthermore, toxic worries of fungal pigments have arisen. Toxicological tests are required depending on the final applications of the pigments in either food, cosmetics, textiles, etc. Leading companies have filed numerous patents for *Monascus*-like pigments and the products are in the market. For centuries, *Monascus* pigments have been used in Asia. Most importantly, red pigments from *P. oxalicum* (Natural Red TM), β-carotene, and lycopene from *Blakesla trispora* have been authorized in Europe.

The use of microbes has tremendous advantages as it does not require petroleum based raw materials and this influences the price of the pigment. The qualitative and quantitative research on fungal pigments should be intensified with negative ecological impact. Hence, there is a necessity to explore novel pigments producing fungi from different taxonomic groups to meet the existing demand for natural pigments. The chemical diversity of fungi should be explored for identifying the potent pigments and toxicological testing must be carried out to be accepted by consumers.

## 9. Conclusions

The growing universal concern is promoting the needs of eco-friendly natural dyes from sustainable resources to counter the hazardous effects of synthetic colorants on the environment and human health. In recent times, the potential of fungal pigment as one of the microbial pigments is explored for textile dyeing in addition to various other applications. The fungi produced an extraordinary range of pigments from different chemical classes. Fungal pigments proved to be nontoxic and achieved adequate color stability to withstand temperature, pH, and additives with color fastness properties when applied to textile fabrics. The advances in biotechnology, genetic engineering strategies for strain improvement, and immense fungal diversity have boosted the use of fungal pigments in the textile industry. However, for commercial applicability, fungal pigments should be tested for toxicity and quality to obtain regulatory approval before entering the market.

## Figures and Tables

**Table 1:** Fungal pigments and their application in the textile industry.

Fungi	Pigment	Color	Fabrics	References
*Penicillium oxalicum*	Anthraquinones	Arpink Red	Wool	Sardaryan et al. [97]
*Trichoderma virens*	Anthraquinones	Yellow	Silk, Wool	Sharma et al. [77]
*Alternaria alternata*	Anthraquinones	Reddish-Brown		
*Curvularia lunata*	Anthraquinones	Black		
*Alternaria alternata*	Anthraquinones	Reddish-Brown	Cotton	Devi and Karuppan [95]
*Thermomyces sp.*	Anthraquinones	Yellow	Cotton, Silk, Wool	Poorniammal et al. [94]
*Trichoderma sp.*	Anthraquinones	Yellow	Cotton, Silk, Silk cotton	Devi [98]
*Trichoderma sp.*	Anthraquinones	Yellow	Silk, Wool	Gupta et al. [93]
*Penicillium oxalicum* (NRC M25)	Anthraquinones	Faint Reddish- Brown	Wool	Mabrouk et al. [24]
*Sclerotinia sp.*	Anthraquinones	Pinkish-Red	Cotton	Perumal et al. [84]
*Aspergillus sp.* AN01	Asperyellone	Yellow	Silk, Cotton, Synthetic and Wool fabrics	Iswarya et al. [99]
*Monascus purpureus*	Azaphilones	Red	Cotton	Velmurugan et al. [96]
*Penicillium purpurogenum*		Yellow		
*Isaria farinosa*		Pink		
*Fusarium verticillioides*		Reddish- Brown		
*Emericella nidulans*		Red		
*Penicillium murcianum*	Carotenoids	Yellow	Wool	Hernandez et al. [100]
*Talaromyces australis*		Red		
*Talaromyces australis*	2, 4-Di-tert-butylphenol	Red	Cotton fabric	Shibila and Nanthini [101]
*Phoma herbarum*	Magenta pigment	Magenta	Nylon	Chiba et al. [102]
*Monascus purpureus*	Monascorubramine	Red	Wool	De santis et al. [103]
*Talaromyces verruculosus*	Polyketide	Red	Cotton fabric	Chadni et al. [89]
*Monascus purpureus*	Rubropunctamine	Red	Wool	De santis et al. [103]
*Chlorociboria aeruginosa*	Quinones	Green	Bleached cotton, Spun polyamide, Spun polyester, Spun polyacrylic, Worsted wool	Weber et al. [48]; Hinsch et al. [91]
*Scytalidium cuboideum*		Red		
*Scytalidium ganodermophthorum*		Yellow		
*Aspergillus sp.*	Quinones	Brown	Cotton, Silk, Silk cotton	Devi [98]
*Alternaria alternata*	Quinones	Reddish-Brown	Cotton	Gokarneshan [104]
*Acrostalagmus* (NRC 90)	Quinones	Brown	Wool	Mabrouk et al. [24]
*Alternaria alternata* (NRC17)		Reddish-Brown		
*Alternaria sp.* (NRC 97)		Brown		
*Aspergillus niger* (NRC 95)		Brown		
*Bisporomyces sp.* (NRC 63)		Deep Brown		
*Cunninghamella* (NRC 188)		Faint Reddish-Brown		
*Penicillium chrysogenum* (NRC 74)		Deep Brown		
*Penicillium italicum* (NRC E11)		Brown		
*Penicillium regulosum* (NRC 50)		Brown		
*Phymatotrichum sp.* (NRC 151)		Reddish-Brown		
